# Hydronephrosis Resulting from Bilateral Ureteral Stenosis: A Late Complication of Polyoma BK Virus Cystitis?

**DOI:** 10.1155/2010/297358

**Published:** 2010-09-27

**Authors:** N. Basara, F.-M. Rasche, T. Schwalenberg, C. Wickenhauser, M. Maier, J. Ivovic, D. Niederwieser, T. H. Lindner

**Affiliations:** ^1^Department of Hematology/Oncology and Hemostaseology, University Hospital Leipzig, Johannisallee 32a, 04103 Leipzig, Germany; ^2^Department of Nephrology, University Hospital Leipzig, 04103 Leipzig, Germany; ^3^Department of Urology, University Hospital Leipzig, 04103 Leipzig, Germany; ^4^Institute of Pathology, Faculty of Medicine, University of Leipzig, Leipzig, Germany; ^5^Institute of Virology, University Hospital Leipzig, 04103 Leipzig, Germany; ^6^Department of Urology, General Hospital, Bar, Montenegro

## Abstract

We report here a case of acute lymphoblastic leukemia in remission presenting a late-onset bilateral hydronephrosis probably due to polyoma BK virus-induced proliferation of bladder endothelium on both ostii. The diagnosis was made virologically by BK virus Polymerase Chain Reaction (PCR) detection in the absence of any other bladder disease. Awareness of this late complication is necessary not only in patients after renal transplantation but also in patients after hematopoietic stem cell transplantation from matched unrelated donor.

## 1. Introduction

Polyomavirus BK was first isolated 1971 from the urine of an asymptomatic renal transplant patient who was hospitalized with acute renal injury and with initials BK [1]. It is a deoxyribonucleic acid (DNA) virus with circular double-stranded DNA genome and a nonenveloped icosahedral capsid [2]. Primary infection with BK virus usually occurred in childhood and was generally asymptomatic. After primary infection, BK virus entered a latent state and resided in uroepitel cells [3]. More than 80 percent of adults had serologic evidence of infection [3]. In immunocompromised hosts, such as renal transplant patients, BK virus may reactivate and cause hemorrhagic cystitis and severe allograft dysfunction with acute tubulointerstitial nephritis [4, 5]. BK viruria has been associated with a variety of clinical manifestations, mostly with hemorrhagic cystitis, being associated with significant morbidity and mortality [6, 7]. Importantly, BK virus infection could escalate from viruria to viremia to nephropathy [8]. BK nephropathy began as a localized viral presence in the tubular epithelial cells of the kidney and progressed to a diffuse and destructive T-cell-mediated interstitial nephritis [9].

About 50% of patients after hematopoietic bone marrow transplantation presented with BK viruria, usually within 2 months of transplantation [10, 11], with similar incidence in both autologous and allogenic recipients (39–54%) [12]. 

## 2. Case Report

**Figure 1:**
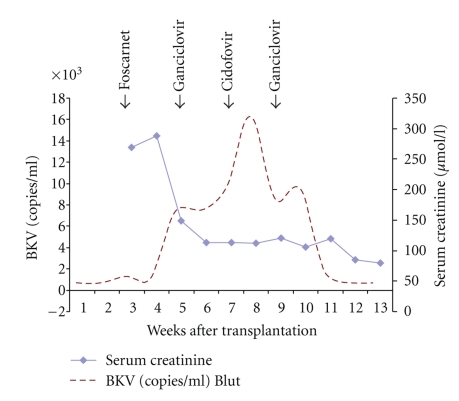
BK virus (BKV) levels in a patient after MUD-PBSCT. Brocken lines indicate BKV in plasma and diamonds serum creatinine level. Horizontal arrows indicate changes in the antiviral therapy.

A 18-year-old patient with a history of Matched-Unrelated-Donor Peripheral Blood Stem Cell Transplantation (MUD-PBSCT) was admitted to the hospital because of renal failure. In November 2007 the patient was first diagnosed with acute lymphoblastic leukemia of T cells (T-ALL) at another institution, where he was treated with induction therapy according to Hyper-CVAD Protocol (Cyclophosphamide 300 mg/m^2^ intravenously (IV) over 3 hours every 12 hours for six doses on days 1 through 3, with mesna given by continuous infusion, vincristine 2 mg IV days 4 and 11, doxorubicin 50 mg/m^2^ IV day 4, and dexamethasone 40 mg daily on days 1 through 4 and 11 through 14) and was refractory when he presented in our department of hematology/oncology [13]. The diagnosis of T-ALL has been confirmed, immunophenotype precursor T-ALL (CD7, CD3, CD2, CD4, and CD8 pos), cytogenetic 46, XY, Fluorescein in sito hybridisation (FISH) analysis 87% deletion of p16 on the chromosome 9 region p21. He was then treated with induction therapy according to the German multicenter ALL protocol (GMALL) achieving only reduction of blasts after Induction II. Briefly, the induction in GMALL protocol was composed of eight cytotoxic drugs administered sequentially in two phases over an 8-week period. During the first 4 weeks (phase I), the patient received 60 mg/m^2^ prednisolone PO daily (days 1 through 28) plus 45 mg/m^2^ daunorubicin and 2 mg vincristine IV weekly (days 1, 8, 15, and 22). L-asparaginase (5 000 U/m^2^) was administered IV daily (days 15 through 28). In the second 4 weeks (phase II), the patient received two doses of 650 mg/m^2^ cyclophosphamide IV (days 29, 43, and 57) together with 60 mg/m^2^ 6-mercaptopurine PO daily (days 29 through 57) and four courses of 75 mg/m^2^ cytarabine IV (days 31 through 34, 38 through 41, 45 through 48, and 52 through 55) [14]. He was then treated with nelarabine and achieved complete remission (CR1) following MUD-PBSCT. The conditioning consisted of total body irradiation (TBI), 12 Gy, given fractionated 2 times daily for three days, from day −6 to day −4, and cyclophosphamide 60 mg/kg once daily i.v. on days −3 and −2 (total dose 120 mg/kg b.w.). A Human Leukocyte Antigen (HLA) identical unrelated donor peripheral blood stem cells were infused on day 0. Graft-versus-Host Disease (GvHD) prophylaxis consisted of thymoglobulin (2 mg/kg b.w. daily, from day −3 to −1, total dose of 6 mg/kg b.w.), cyclosporine 5 mg/kg starting on day −1, and methotrexate at days +1, +3, +6, and +11 intravenously 15 mg absolute. The bone marrow puncture on day +28 showed a complete hematological remission with 100% donor chimerism. Along with the regeneration of white blood cells the patient developed a methylprednisolone-dependent GvHD of the skin and gastrointestinal, grade II. After 10 days the therapy with corticosteroids could be discontinued by rapid improvement of the GvHD-related symptoms. Under the therapy with corticosteroids the patient developed a herpes simplex related oesophagitis on day 21 post transplant which was treated with 30 g daily over 5 days i.v. immunoglobulin and foscarnet 90 mg/kg b.w. divided in 2 doses. On day +28 after PBSCT the patient developed a hemorrhagic cystitis with high urine titers of BK polyoma virus and CMV infection. A therapy with ganciclovir was initiated for ten days. Under treatment with ganciclovir, CMV infection improved, and the treatment with cidofovir was started (1 mg/kg b.w. for two weeks). The BK Virus viruria and viremia improved, and the patient received ganciclovir treatment for additional two weeks (Figure 1). The cyclosporine level was from 200 to 150 from day 0 to day 30, followed with level of 100 from day 30 to day 40 post transplant, and level of 50 from day 40 to day 50. The cyclosporine has been discontinued on day +70 after MUD-PBSCT in absence of acute GvHD and as recommended in GMALL protocol. On day +48 after PBSCT a cystoscopy with a continuous intravesical saline irrigation was started and discontinued 2 weeks later. The patient symptoms improved, and creatinine levels returned to normal. He was discharged from hospital; his medications at the time of discharge included ciprofloxacin (250 mg twice daily), valacyclovir (500 mg twice daily), fluconazole (200 mg daily), folate, and cotrimoxazole twice weekly for prophylaxis of pneumocystis carinii infection.

Over the next year the levels of urea nitrogen and creatinine gradually rose (Table 1), which was attributed to drug toxicity. cotrimoxazole prophylaxis was stopped. Cyclosporine was discontinued since no signs of GvHD were present 6 months after PBSCT. The serum creatinine level was 153 *μ*mol/l (Table 1). Routine urinary diagnostic measures were carried out and showed inconsistent microhematuria. BK viruria was negative in this period. 

On admission, blood pressure was 130/80 mmHg with 90 heart beats per minute, a temperature of 36.4°C, and an oxygen saturation of 99% when the patient was breathing ambient air. The lungs were clear, the results of the remainder of the examination were normal. The complete blood count and levels of serum electrolytes, liver function tests, and levels of total protein and albumin were normal. The urine sediment was not nephritic but contained a small amount of leukocytes indicating an inflammatory process such as cystitis. Results of other laboratory tests are shown in Table 1. 

**Figure 2:**
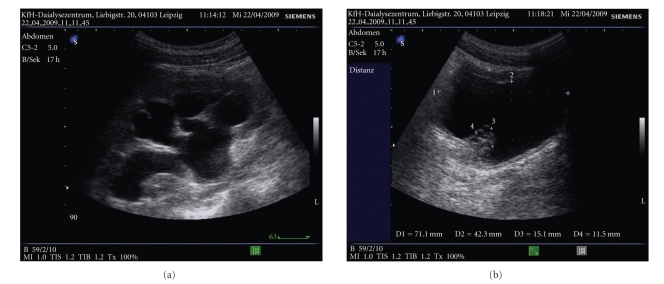
Ultrasound examination of the right kidney and the bladder on admission. Hydronephrosis stage III-IV with severely reduced renal parenchyma (a) and bladder tumorous formation (b).

Ultrasonographic examination of the kidneys showed bilateral hydronephrosis stage III-IV (Figure 2(a)). In addition, decreased echotexture bladder tumours were seen (Figure 2(b)). The patient was presented to the urologist. Cystoscopy with urethrography has shown exophytic bladder tumours in both ostii. The extirpation of the tumours was performed and sent off for pathological examination. In addition a double-J catheter was placed. The hydronephrosis of the left kidney improved immediately and improved to grade II in the right kidney. For histological examination tissues were formalin-fixed overnight and embedded in paraffin. Hematoxylin & Eosin staining of 5 micrometer sections was performed following standard procedures. 

**Figure 3:**
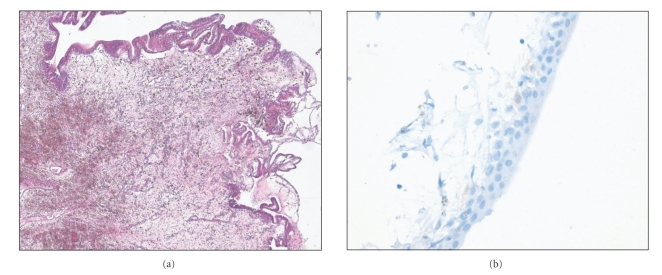
Urothelial mucosa with erosions, edema, fresh bleedings, and bleeding residues. Moderate inflammatory reaction (a). Immunohistochemically, large T antigen could not be visualized (b) ((a) magnification ×100, (b) magnification × 400).

The pathohistology of the specimen showed a chronic cystical urocystitis with bleedings (Figure 3(a)). Tissue probes of both ureters were stained for SV40 large T antigen (clone pAb416, Oncogene Science, Boston, Mass, USA; dilution 1 : 100, antigen retrieval and amplification with catalyzed system amplification, Dako, Carpinteria, CA). The reactions were detected by means of the streptavidin-biotin method and were revealed using diaminobenzidine as a chromogen [15, 16]. In all tissue probes immunohistochemistry for large T antigen was negative (Figure 3(b)). 

The patient has undergone double-J catheter controls and changes every two months and is doing well 2 years after PBSCT and 1 year after operative extirpation of tumours. The last control was in April 2010 (Table 1).

## 3. Detection of BK Virus by Real Time PCR

Two different regions of the BKV genome were amplified by PCR, VP2 in serum and urine, and large T antigen from tissue biopsies.

Nucleic acids were extracted from 200 microliter blood or urine using magnetic beads (MagNA Pure LC total NA isolation kit, Roche, Mannheim, Germany) according to the manufacturer's instructions while from formalin-fixed, paraffin-embedded material nucleic acids were extracted using the QIAamp DNA FFPE Tissue Kit (Qiagen, Hilden, Germany). VP2 primers lead to amplification of a 131 bp fragment [17]. PCR reaction was carried out in 20 microliter capillaries containing master mix (6 mM MgCl_2_, 0.1 mg/ml BSA (Sigma, St. Louis, USA)) and 10 picoM of each primer (Metabion, Martinsried, Germany), 3 picoM of each hybridization probe (5′-GGCTGCTGCTGCCACAGGATTTTC-FL (probe 1) and 5′LC Red640-GTAGCTGAAATTGCTGCTGGAGAG-ph (probe 2), 0.25 mM dNTP's (Roche)), and 1U Platinum Taq Polymerase (Invitrogen, Karlsruhe, Germany). Target DNA was added in a volume of 5 microliters. Two negative and 4 positive controls in different concentrations were included into each run performed on a LightCycler 1.0 instrument (Roche, Mannheim, Germany) under the following conditions: initial denaturation and activation of hot-start enzyme at 95°C for 30 s, followed by 45 cycles of denaturation at 95°C for 0 s, annealing of primers at 58°C for 10 s, and primer extension at 72°C for 20 s. Fluorescence was monitored by single acquisition during the annealing phase of each PCR cycle and channel setting F2/F1 (640 nm/530 nm). Differentiation of BK virus amplicons from JC virus depends on the binding of the probe 2, which perfectly matches JC virus but differs at the two underlined positions from BK virus. Thus binding to BK virus results in a shifted melting curve (61.5°C for BK versus 63.8°C for JC).

For detection of the viral large T-antigen, a 128 bp long fragment was amplified using modified primers from [18] that match BKV genotypes 1 to 4, forward primer 5′-CAGGCAAGGGTTCTATTACTAAAT-3′, reverse primer 5′-GCAACAGCAGATTCYCAACA-3′, probe 5′-6FAM-AAACTGGTGTAGATCAGARGGAAAGTCTTTAGGGTCTT-BBQ. 

PCR conditions were identical to the one described above except TaqMan probe that was used at 4 pico molar, primer annealing was 56°C for 10 s, and fluorescence acquisition was at the end of each primer extension phase at 530 nm.

## 4. Discussion

We described a young patient with precursor T-ALL who had received an unrelated matched donor PBSCT and one year later presented with increase creatinine levels and bilateral hydronephrosis. 

Nephrological and urological examination showed an epithelial proliferation of bladder on both ostii being responsible for bilateral hydronephrosis. Further pathological and molecular analysis revealed widespread endothelial infection by a BK virus. We speculate that the endothelial cell injury led to capillary injury, oedema, and tumorous proliferation on both ostii. Other organs were not involved. 

This case demonstrated a very late (one year after MUD-HSCT) proliferative endothelial infection by a virus related to severe viral load and hemorrhagic cystitis 4 weeks after unrelated PBSCT. Although the tumour mass could not be unambiguously attributed to BKV since the staining of large T antigen was negative in immunohistochemical analysis, large T-cell antigen and BK virus were positive by PCR, thus supporting the view that BK virus may indeed play a role in the development of this tumour. The oncogenic potential of BK virus has been well recognized [2, 3]; Geetha et al. reported the case of a simultaneous pancreas and kidney transplant recipient who developed BKV interstitial nephritis and carcinoma of the bladder with widespread metastasis. For both the primary tumour and the metastasis, immunohistochemical staining for the large T-cell antigen showed strong nuclear positivity [19]. However, the diagnosis of the role of BK virus infection as a transient “hit and run” agent is according to Hirsch [20] difficult to prove with the current diagnostic tools. 

BK virus is an important pathogen and cause of nephropathy in renal transplant recipients, but its significance following PBSCT is less well described. Recently, O'Donnell et al. have shown in 124 allogeneic PBSCT patients that BK viruria was manifest in 65% of the patients; 17% of the patients developed viremia. The median time from transplantation to BK viremia development was 128 days and was longer for viruria (24 days) [21]. Among clinical factors such as sex, disease, transplant type, alemtuzumab use, cytomegalovirus viremia, graft-versus-host disease, and donor HLA C7 allele, only CMV viremia was more common in patients with BK virus infection. In addition, BK virus infection was analysed with other clinical factors related to the development of post-PBSCT renal impairment. On multivariate analysis, only BK viremia and alternative donor transplantation were independent predictors of development of post-HSCT renal impairment [21]. There are numerous papers showing that there is no association between CMV and BKV reactivation. One of the best examples is by Hirsch et al., 2002 NEJM, where no CMV prophylaxis was given and only CMV seropositive and BKV seropositive kidney recipients were examined [22]. Harkensee et al. published the results on the incidence of BK virus in 102 children who underwent allogeneic HSCT [23]. BK virus DNA was detected in the urine of 47% children; the only significant factor for hemorrhagic cystitis was high-dose chemotherapy. The treatment included the use of cidofovir, which was feasible and without significant toxicity [23]. The evidence-based guidelines for clinical management include in addition to virus-specific therapy the use of estrogen or bladder instillation with alum salts or prostaglandin, as recently reviewed [24–26]. 

The chronic renal failure was a multifactorial process. The transforming potential of BK virus infection has been noted early both in vitro culture models and in vivo. One of the patients with BK virus-associated nephropathy after kidney transplantation reported by Vats et al. [26] presented with an echogenic renal mass that resolved with immunosuppression reduction and treatment with cidofovir. Contributing factors included drug toxicity around PBSCT (cyclosporine in particular), polyoma BK viruria, although it was not proven whether the kidneys were involved, and chronic hydronephrosis along with an inflammatory process due to the urine congestion.

In summary, we could show an unusual case of late onset of bilateral hydronephrosis probably due to BK virus-induced tumorous bladder proliferation on both ostii in a young patient after MUD-PBSCT, successfully treated with operative incision and DJ catheter.

## Figures and Tables

**Table 1:** Results of laboratory tests.

Variable	Reference range in adults*	Before MUD-PBSCT	1 month after MUD-PBSCT	6 months after MUD-PBSCT	On admission	1 year later
Urea nitrogen (mmol/l)	3.8–8.0	6.9	33.3	5.8	21.7	12
Creatinine (*μ*mol/l)	62–106	64	233	153	299	150
Cyclosporine (ng/ml)			128			
eGFR (MDRD; ml/min)	>90^#^	137^#^	31	50	23	51
24-Hr urine collection						
Total Volume (ml)		2650	6650	3200	4200	
Creatinine (mg/ml)		3865	1378	5660	2812	
Total Protein (mg/24 h)	0–135	Negative	1390	Negative	382	Negative

*The ranges used at University Hospital Leipzig are for adults who are not pregnant and do not have medical conditions that could affect the results.

This is only for visualization. The eGFR MDRD formula has not been validated outside the 20 to 60 ml/min range.
